# Stentless vs. stented bioprosthesis for aortic valve replacement: A case matched comparison of long-term follow-up and subgroup analysis of patients with native valve endocarditis

**DOI:** 10.1371/journal.pone.0191171

**Published:** 2018-01-16

**Authors:** Andreas Schaefer, Jannis Dickow, Gerhard Schoen, Sumi Westhofen, Lisa Kloss, Tarik Al-Saydali, Hermann Reichenspurner, Sebastian A. Philipp, Christian Detter

**Affiliations:** 1 Cardiovascular Surgery, University Heart Center Hamburg, Hamburg, Germany; 2 Department of Medical Biometry and Epidemiology, University Medical Center Hamburg-Eppendorf, Hamburg, Germany; 3 Department of Cardiology and Intensive Care Medicine, Elbe Clinic Stade, Stade, Germany; University of Bern, University Hospital Bern, SWITZERLAND

## Abstract

**Background:**

Current retrospective evidence suggests similar clinical and superior hemodynamic outcomes of the Sorin Freedom Solo stentless aortic valve (SFS) (LivaNova PLC, London, UK) compared to the Carpentier Edwards Perimount stented aortic valve (CEP) (Edwards Lifesciences Inc., Irvine, California, USA). To date, no reports exist describing case-matched long-term outcomes and analysis for treatment of native valve endocarditis (NVE).

**Methods:**

From 2004 through 2014, 77 consecutive patients (study group, 59.7% male, 68.9 ± 12.5 years, logEuroSCORE II 7.6 ± 12.3%) received surgical aortic valve replacement (SAVR) with the SFS. A control group of patients after SAVR with the CEP was retrieved from our database and matched to the study group regarding 15 parameters including preoperative endocarditis. Acute perioperative outcomes and follow-up data (mean follow-up time 48.7±29.8 months, 95% complete) were retrospectively analyzed.

**Results:**

No differences in early mortality occurred during 30-day follow up (3/77; 3.9% vs. 4/77; 5.2%; p = 0.699). Echocardiographic findings revealed lower postprocedural transvalvular pressure gradients (max. 17.0 ± 8.2 vs. 24.5 ± 9.2 mmHg, p< 0.001/ mean pressure of 8.4 ± 4.1 vs. 13.1 ± 5.9 mmHg, p< 0.001) in the SFS group. Structural valve degeneration (SVD) (5.2% vs. 0%; p = 0.04) and valve explantation due to SVD or prosthetic valve endocarditis (PVE) (9.1% vs. 1.3%; p = 0.04) was more frequent in the SFS group. All-cause mortality during follow-up was 20.8% vs. 14.3% (p = 0.397). When patients were divided into subgroups of NVE and respective utilized bioprosthesis, the SFS presented impaired outcomes regarding mortality in NVE cases (p = 0.031).

**Conclusions:**

The hemodynamic superiority of the SFS was confirmed in this comparison. However, clinical outcomes in terms of SVD and PVE rates, as well as survival after NVE, were inferior in this study. Therefore, we are reluctant to recommend utilization of the SFS for treatment of NVE.

## Introduction

Surgical aortic valve replacement (SAVR) utilizing stentless bioprostheses has been reported a reasonable alternative to stented xenovalves. Main advantages of stentless valves are considered superior hemodynamic outcomes in terms of postoperative transprosthetic pressure gradients and effective orifice area (EOA). Especially in small aortic annuli stentless valves are able to generate adequate EOA and avoid patient-prosthesis mismatch due to the supra-annular implantation technique [1]. However, there is a lack of knowledge regarding clinical long-term outcomes compared to stented bioprostheses [2]. At least, there are few studies emphasizing the risk of premature structural valve deterioration (SVD) and high explantation rates of stentless bioprostheses [1; 3; 4]. Furthermore, there are only few reports for efficacy of treatment for native valve endocarditis (NVE) utilizing stentless bioprostheses in aortic position. NVE remains a life-threatening condition and surgical treatment of NVE is indicated when heart failure due to valve insufficiency or uncontrolled infection occurs, or when embolism due to persistent vegetations >15 mm is anticipated [5; 6]. Beside debridement of infected tissue and repair of cardiac structures utilizing pericardium, SAVR is the operative approach of choice for the infected aortic valve (AV) [7]. For SAVR mechanical and biological prostheses or homografts can be taken into consideration [8; 9]. Recently, SAVR with the Medtronic Freestyle (Medtronic Inc., Minneapolis, MN, USA) stentless bioprosthesis for extensive NVE with aortic root involvement and periannular abscess formation was described with good late survival and low rates of recurrence of prosthetic valve endocarditis (PVE) [10]. One of the most frequently utilized stentless valve is the bovine pericardial Sorin Freedom Solo (SFS) (LivaNova PLC, London, UK). The SFS was reported to present an equivalent safety profile compared to stented bioprostheses, while yielding superior mid- and long-term hemodynamic outcomes in a non-infective setting [11]. Furthermore, the SFS is considered to facilitate more rapid left ventricular (LV) reverse remodeling [12].

We hereby aimed to analyze long-term outcomes of the SFS with regards to mortality, rates of SVD, valve explantation, PVE, and hemodynamic outcomes compared with the Carpentier Edwards Perimount (CEP) stented bovine pericardial aortic valve (Edwards Lifesciences Inc., Irvine, California, USA) in a case-matched study design. Furthermore, a subgroup of patients treated for AV- NVE was evaluated.

## Materials and methods

### Patients

All research was approved by the authors' Institutional Review Board (University Hospital Hamburg-Eppendorf). All clinical investigation has been conducted according to the principles expressed in the Declaration of Helsinki. Informed consent, was obtained from the participants. A consecutive series of 77 patients received SAVR using the stentless bovine pericardial SFS for treatment of severe symptomatic calcified aortic stenosis or aortic regurgitation in cases of NVE (study group). Of those 24.7% (19/77 pts.) suffered from NVE. Allocation of patients to SAVR followed current international recommendations after consensus of the local dedicated heart team [13]. For comparative assessment, a matched control group of 77 patients treated by SAVR using the stented bovine pericardial CEP was retrieved from our dedicated hospital database containing a total of 614 CEP patients. Follow-up was conducted by mail for patients and the treating physicians, respectively. In cases of missing answers, patients and/or physicians were contacted by phone.

### Diagnostic work-up and study procedure

Diagnostics and procedures followed institutional routines: By routine, all patients received preoperative transthoracic echocardiography (TTE) and transesophageal echocardiography in cases of suspected NVE for evaluation of cardiac functional status, valve morphology and assessment of vegetations and/or involvement of the aortic annulus and surrounding structures in NVE. The SFS was preferred in aortic annuli with a diameter ≤ 20 mm; destroyed aortic annuli in NVE, and severly hypertrophed LV.

### Operative technique

All operations were performed through a median sternotomy (in all NVE cases) or partial sternotomy (11.6%; 18/154 pts.) with CPB on the arrested heart using Bretschneider Cardioplegia. SFS valves were implanted in a supra-annular position with a single running Prolene (Ethicon Inc., Somerville, NJ, USA) suture line and interrupted u-stitches for the CEP. All patients received lifelong aspirin as antiplatelet inhibitor.

### Statistics

Baseline, intraprocedural and follow-up data (mean follow-up time 48.7±29.8 months) were collected, entered into a dedicated standardized database and retrospectively analyzed. Primary clinical endpoints were (1) death, (2) re-operation, (3) PVE and (4) structural valve deterioration. Secondary endpoint was hemodynamic performance in terms of (1) peak/mean transprosthetic pressure gradients and (2) trans- and/or paravalvular leakage.

Data are presented as absolute numbers and percentages for categorical variables and mean values and standard deviation for continuous variables unless stated otherwise.

Matching was performed as previously described [14]: To evaluate the effect of a treatment in a non-randomized setting, 1:1 matching (drawing without replacement) was conducted by logistic regression and nearest neighbor matching as the measure of proximity. In a first step matching pairs of all complete cases from the treatment group were identified for the following 15 variables: age, gender, NVE, logEuroSCORE II, New York Heart Association (NYHA) functional class, left ventricular ejection fraction (LVEF), pulmonary hypertension, peripheral artery disease, creatinine at baseline, chronic obstructive pulmonary disease (COPD) > Gold II, previous sternotomy, previous stroke, coronary artery disease (CAD), diabetes mellitus and arterial hypertension. In consecutive steps, all remaining pairs were identified in case of missing data. All computation was carried out by the statistical software R and the R-package MatchIt [15; 16]. Due to the dependence structure of the matched pairs data, we used t-tests the for continuous data and McNemar`s tests for categorical data. A level of significance was set to two-tailed *p* < 0.05.

To further evaluate survival of the study and control group we performed a multivariate COX-regression including five different variables.

## Results

### Baseline demographics and matching results

77 consecutive patients (study group) received SAVR using the SFS valve (study group, 59.7% male, 68.9 ± 12.5 years, logEuroSCORE II 7.6 ± 12.3%). Matching yielded a control group of 77 patients receiving SAVR utilizing the CEP valve who were similar to the study group with regard to 15 important baseline parameters including endocarditis (24.7% vs. 20.1%; p = 0.53]), EuroSCORE II (7.6 ± 12.3 vs. 5.2 ± 6.3; p = 0.09) and Creatinine (1.14 ± 0.61 mg/dl vs. 1.02 ± 0.34 mg/dl; p = 0.08). No significant inter-group differences were present after matching. Detailed patient demographics are summarized in **Table 1**.

**Table 1:** Baseline demographics and matching results.

	Freedom Solo (n = 77)	Edwards Perimount (n = 77)	p-value
Age, y	68.9 ± 12.5	67.1 ± 12.2	0.302
Male gender, % (n)	59.7 (46)	68.8 (53)	0.239
Endocarditis, % (n)	24.7 (19)	20.1 (16)	0.534
EuroSCORE II, %	7.6 ± 12.3	5.2 ± 6.3	0.093
NYHA ≥ III, % (n)	56.2 (44)	58.4 (45)	0.870
PHT > 60 mmHg, % (n)	16.9 (13)	11.7 (9)	0.836
Extracardiac atheropathy, % (n)	11.7 (9)	7.8 (6)	0.685
- Peripheral artery disease	2.6 (2)	1.3 (1)	
- Carotid artery stenosis	7.8 (6)	6.5 (5)	
- Both	1.3 (1)	0.0 (0)	
Creatinine, mg/dl	1.14 ± 0.61	1.02 ± 0.34	0.083
COPD > GOLD II, % (n)	9.1 (7)	7.8 (6)	0.772
Previous cardiac surgery, % (n)	9.1 (7)	10.4 (8)	0.786
Prior stroke, % (n)	3.9 (3)	5.2 (4)	0.690
Coronary heart disease, % (n)	36.4 (28)	33.8 (26)	0.962
- 1-VD	14.3 (11)	14.3 (11)	
- 2-VD	10.4 (8)	10.4 (8)	
- 3-VD	11.7 (9)	9.1 (7)	
Diabetes, % (n)	19.5 (15)	13.0 (10)	0.527
Arterial hypertension, % (n)	59.7 (46)	62.3 (48)	0.619

EuroSCORE: European System for Cardiac Operative Risk Evaluation, NYHA: New York Heart Association, PHT: Pulmonary hypertension, COPD: Chronic obstructive pulmonary disease, GOLD: Global Initiative For Chronic Obstructive Lung Disease, VD: Vessel disease

### Perioperative data

There were no significant differences between SFS and CEP groups regarding baseline echocardiography parameters, with the exception of preoperative peak pressure gradients, which were significantly lower in the SFS group (55.5 ± 29.7 mmHg vs. 71.2 ± 29.3 mmHg, p = 0.004), preoperative EOA (1.0 ± 0.5 cm^2^ vs. 0.8 ± 0.5 cm^2^, p = 0.044) and diameter of the interventricular septum (IVS) (12.0 ± 2.2 mm vs. 14.0 ± 2.9 mm, p = 0.015). For detailed echocardiography values see **Table 2**.

**Table 2:** Preprocedural echocardiography.

	Freedom Solo (n = 77)	Edwards Perimount (n = 77)	p-value
Peak gradient (mmHg)	55.5 ± 29.7	71.2 ± 29.3	0.004
Mean gradient (mmHg)	33.9 ± 20.5	41.0 ± 18.0	0.052
AVA (cm^2^)	1.0 ± 0.5	0.8 ± 0.5	0.044
Aortic valve regurgitation ≥ Grade III, % (n)	23.4 (18)	23.4 (18)	0.805
Mitral valve regurgitation ≥ Grade III, % (n)	9.1 (7)	11.7 (9)	0.575
LV EF < 45%, % (n)	11.7 (9)	15.6 (12)	0.590
Diameter (mm)			
- LVEDD	56.2 ± 9.5	53.2 ± 11.6	0.360
- LVESD	31.6 ± 5.9	38.4 ± 10.8	0.100
LV hypertrophy, % (n)	41.6 (32)	55.8 (43)	0.325

AVA: Aortic valve area, LVEF: Left ventricular ejection fraction, LV: Left ventricular, LVEDD: Left ventricular end diastolic diameter, LVESD: Left ventricular end systolic diameter, IVS: Interventricular septum, PW: Posterior wall

Rate of re-do surgery did not differ between SFS and CEP group. In the control group significant more patients presented an acute indication for SAVR (elective: 90.9%, acute: 2.6%, emergency: 6.5% vs. elective: 77.9%, acute: 14.3%, emergency: 7.8%, p = 0.029). Extracorporeal circulation (ECC) time (135.0 ± 48.7 min vs. 132.3 ± 50.4 min, p = 0.76), aortic cross clamp (ACC) time (90.6 ± 37.6 min vs. 90.1 ± 32.7 min, p = 0.94) and rate of concomitant procedures (42.9% vs. 42.9%, p = 1.0) showed no significant differences among groups. In the SFS group a longer hospital stay (12.0 ± 8.4 d vs. 6.2 ± 3.6 d, p< 0.001), a higher rate of blood transfusion (70.1% vs. 33.8%, p< 0.001), and a shorter ventilation time (8.8 ± 11.5 h vs. 14.9 ± 5.8 h, p< 0.001) was documented. Detailed periprocedural data are summarized in **Table 3**.

**Table 3:** Periprocedural data.

	Freedom Solo (n = 77)	Edwards Perimount (n = 77)	p-value
Re-do surgery, % (n)	9.1 (7)	10.4 (8)	0.786
Urgency, % (n)			
- Elective	90.9 (70)	77.9 (60)	0.029
- Acute	2.6 (2)	14.3 (11)	
- Emergency	6.5 (5)	7.8 (6)	
LOS ICU (d)	3.8 ± 7.3	2.5 ± 1.8	0.160
LOS Hospital (d)	12.0 ± 8.4	6.2 ± 3.6	< 0.001
Procedure time (min)	275.7 ± 64.0	281.9 ± 81.5	0.610
Blood transfusion, % (n)	70.1 (54)	33.8 (26)	< 0.001
Blood transfusion, n	4.4 ± 6.4	3.2 ± 2.9	0.260
Access, % (n)			
- Median sternotomy	64.9 (50)	63.6 (49)	0.083
- Minimal invasive	31.2 (24)	29.9 (23)	
Procedure, % (n)			
- Isolated	57.1 (44)	57.1 (44)	1
- Two or more procedures	42.9 (33)	42.9 (33)	
Mitral valve procedure, % (n)			
- Valve repair	13.0 (10)	3.9 (3)	0.137
- Valve replacement	2.6 (2)	3.9 (3)	
Ventilation time (h)	8.8 ± 11.5	14.9 ± 5.8	< 0.001
ECC (min)	135.0 ± 48.7	132.3 ± 50.4	0.760
ACC (min)	90.6 ± 37.6	90.1 ± 32.7	0.940

LOS: Length of stay, ICU: Intensive care unit, ECC: Cardiopulmonary bypass, ACC: Aortic cross clamp

### Echocardiographic follow-up

In the SFS group, peak and mean transvalvular gradients as determined by TTE prior to discharge decreased from 55.5 ± 29.7 mmHg to 17.0 ± 8.2 mmHg and 33.9 ± 20.5 mmHg to 8.4 ± 4.1 mmHg, respectively (both *p*<0.01). Effective orifice area (EOA) increased from 1.0 ± 0.5 cm^2^ to 2.16 ± 0.57 cm^2^ (*p*<0.01) compared to baseline values. Corresponding data in the CEP group were: decrease of peak and mean transvalvular gradients from 71.2 ± 29.3 mmHg to 24.5 ± 9.2 mmHg and 41.0 ± 18.0 mmHg to 13.1 ± 5.9 mmHg (both *p*<0.01), increase of EOA from 0.8 ± 0.5 cm^2^ to 2.07 ± 0.48 cm^2^ (*p*<0.01). The SFS group presented significant lower postoperative peak and mean pressure gradients compared to the CEP group (17.0 ± 8.2 mmHg vs. 24.5 ± 9.2 mmHg (p< 0.001) and 8.4 ± 4.1 mmHg vs. 13.1 ± 5.9 mmHg (p< 0.001)).

There was a significant higher rate of transvalvular leakage (TVL) = Grade I (26.0% vs. 3.9%, p< 0.001) in the SFS group. Rates of paravalvular leakage (PVL) ≥ Grade I were comparable among groups (7.8% vs. 3.9%, p = 0.521).

### Early and late outcome

There were 3/77 deaths (3.9%) during the 30-day follow up in the SFS group and 4/77 (5.2%) in the CEP group (p = 0.699). For detailed acute 30-day outcome data see **Table 4**.

**Table 4:** Clinical 30-day outcome and echocardiographic results at discharge.

	Freedom Solo (n = 77)	Edwards Perimount (n = 77)	p-value
Creatinine (mg/dl)	1.2 ± 0.5	1.0 ± 0.4	0.036
Creatinine peak (mg/dl)	1.6 ± 1.1	1.3 ± 0.6	0.010
AVR ≥ Grade II, % (n)			
- Valvular	0 (0)	0 (0)	0.867
- Paravalvular leak	2.6 (2)	0 (0)	0.521
MVR ≥ Grade II, % (n)	3.9 (3)	9.1 (7)	0.174
AVA (cm^2^)	2.16 ± 0.57	2.07 ± 0.48	0.782
PPR (cm^2^/m^2^)	1.1 ± 0.3	1.0 ± 0.2	0.600
Peak gradient (mmHg)	17.0 ± 8.2	24.5 ± 9.2	< 0.001
Mean gradient (mmHg)	8.4 ± 4.1	13.1 ± 5.9	< 0.001
LVEF < 45%, % (n)	5.2 (4)	7.8 (6)	0.557
Cardiac tamponade, % (n)	9.1 (7)	15.6 (12)	0.624
Wound healing deficit, % (n)	2.6 (2)	1.3 (1)	0.207
MACCE, % (n)	2.6 (2)	0.0 (0)	0.166
- Stroke, TIA	2.6 (2)	0.0 (0)	
- Myocardial infarction	0.0 (0)	0.0 (0)	
Death, % (n)	3.9 (3)	5.2 (4)	0.699
Pacemaker, % (n)	2.6 (2)	0.0 (0)	0.157

NYHA: New York Heart Association, AVR: Aortic valve regurgitation, MVR: Mitral valve regurgitation, AVA: Aortic valve area, PPR: Patient prosthesis ratio, LVEF: Left ventricular ejection fraction

**Fig 1:**
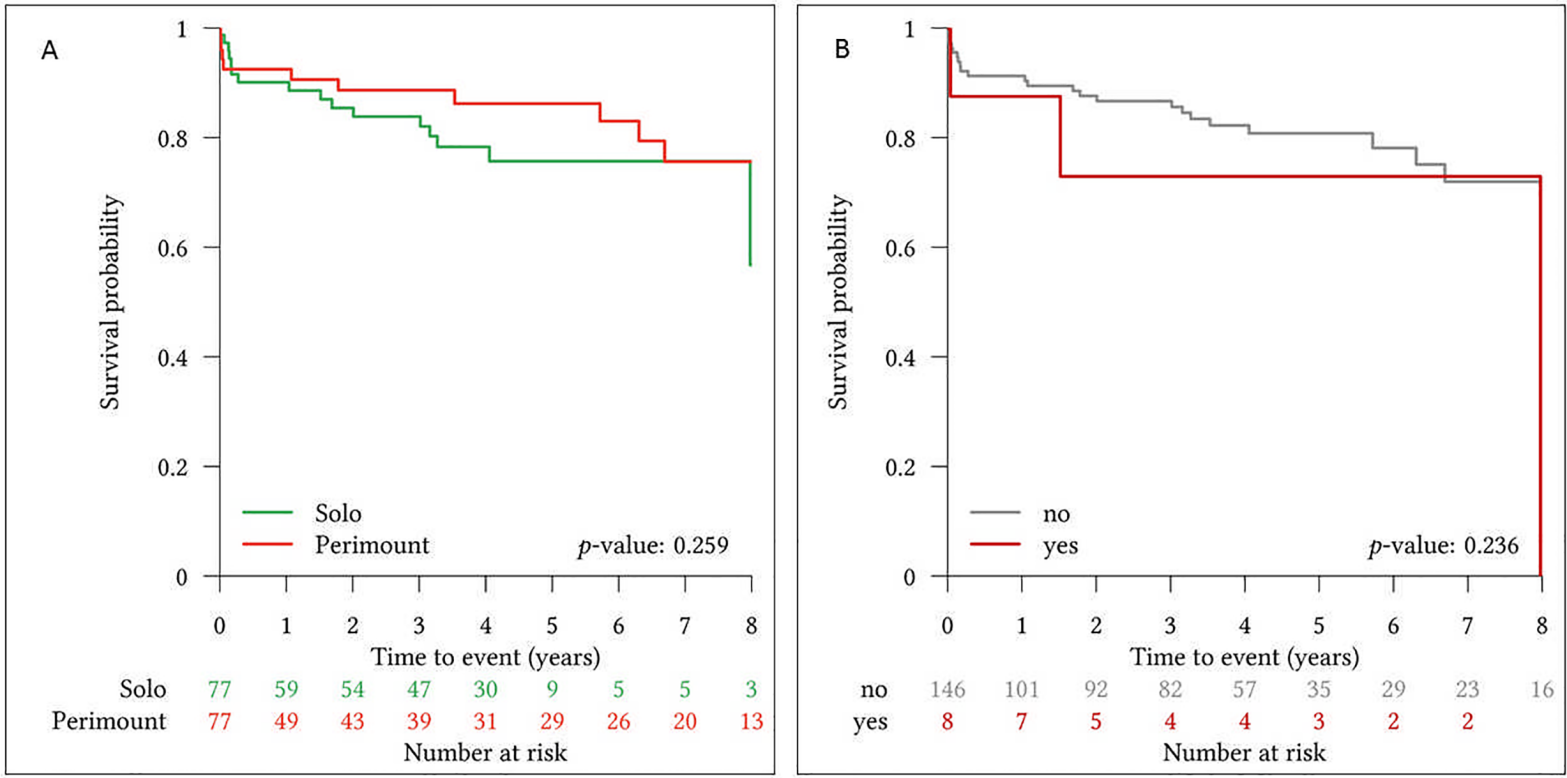
**Kaplan-Meier survival curve for survival comparison of Sorin Freedom Solo and Carpentier Edwards Perimount bioprosthetic heart valves (A) and survival probability for patients with or without re-do surgery after index procedure [no = without re-do surgery; yes = with re-do surgery] (B)**.

All-cause mortality during the mean follow-up of 48.7±29.8 months was 20.8% (16/77) in the SFS group and 14.3% (11/77) in the CEP group, showing no significant difference (p = 0.397). Kaplan-Meier curves for long-term survival showed no significant group differences among groups (p = 0.259) and are presented in **Fig 1A**.

SVD (2 cases of severe calcification with subsequent severe bioprosthetic stenosis, 2 cases of severe paravalvular leakage) (5.2% vs. 0%; p = 0.04) and valve explantation due to SVD and/or PVE (9.1% vs. 1.3%; p = 0.04) was more frequent in the SFS group. Re-do surgery due to SVD and PVE was not associated with a higher risk for death in follow-up (**Fig 1B**). No significant differences in postoperative stroke (5.2% vs. 2.6%, p = 0.507) or myocardial infarction rates (0% vs. 1.3%, p = 0.281) during follow-up were observed. Detailed follow-up data are summarized in **Table 5**.

**Table 5:** Long term follow-up.

	FreedomSolo (n = 77)	Edwards Perimount (n = 77)	p-value
Events, % (n)			
- Valve degeneration	5.2 (4)	0 (0)	0.049
- Thromboembolic	1.3 (1)	0 (0)	0.341
- Valve thrombosis	0 (0)	0 (0)	0.571
- Endocarditis	5.2 (4)	1.3 (1)	0.211
- Paravalvular leak	2.6 (2)	0 (0)	0.176
MACCE, % (n)	5.2 (4)	3.9 (3)	0.456
- Stroke	5.2 (4)	2.6 (2)	0.507
- Myocardial infarction	0 (0)	1.3 (1)	0.281
Explantation/ Re-Do, % (n)	9.1 (7)	1.3 (1)	0.038
- Structural valve deterioration	5.2 (4)	0 (0)	0.285
- Endocarditis	3.9 (3)	1.3 (1)	
Mortality, % (n)			
- Survival	79.2 (61)	85.7 (66)	0.397
- Overall mortality	20.8 (16)	14.3 (11)	
Cause of death, % (n)			
- Cardiac related	3.9 (3)	2.6 (2)	0.937
- Valve related	6.5 (5)	1.3 (1)	0.211
- Sepsis	1.3 (1)	1.3 (1)	0.727
- Multi organ failure	1.3 (1)	0 (0)	0.420
- Tumor	3.9 (3)	2.6 (2)	0.937
- Other	0 (0)	1.3 (1)	0.197
- Unknown	3.9 (3)	5.2 (4)	0.780

MACCE: Major adverse cardiac and cerebrovascular events

**Fig 2:**
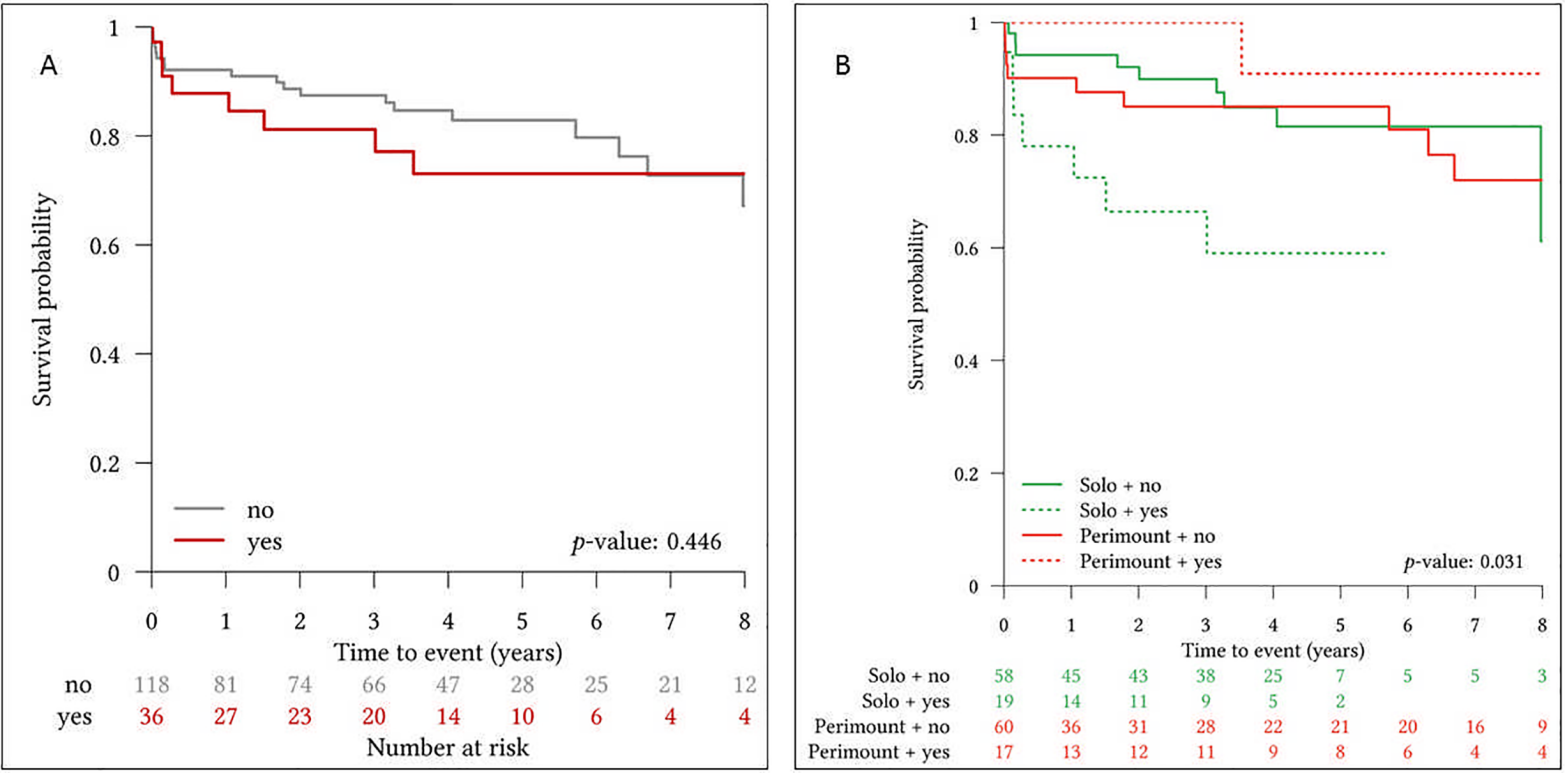
**Survival probability for patients with or without NVE prior to index procedure [no = no preoperative endocarditis; yes = preoperative endocarditis] (A) and survival probability for patients treated with Sorin Freedom Solo or Carpentier Edwards Perimount bioprosthetic heart valves with or without preoperative native valve endocarditis [NVE] [no = no NVE; yes = NVE] (B)**.

When comparing patients with or without preoperative NVE in survival analysis, no group differences in survival were present (**Fig 2A**). However, when patients were divided into subgroups of NVE and respective utilized bioprosthesis, the SFS presented impaired outcomes in NVE cases (p = 0.031) (**Fig 2B**).

Multivariate COX-analysis revealed a pronounced impact of re-do surgery on survival (HR: 7.63, CI: 1.65–35.25, p = 0.009). Also, age and preoperative NVE were connected with an increased risk of death during follow up with hazard ratios of 2.23 and 2.56, respectively.

**Fig 3:**
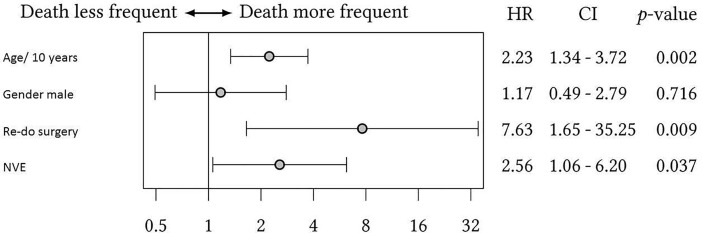
Forest-plot of multivariate COX-analysis with inclusion of four parameters; NVE native valve endocarditis.

Details of multivariate COX-analysis are depicted by Forest-plot (**Fig 3**).

## Discussion

### Main findings

The hemodynamic superiority of the SFS in terms of postoperative transprosthetic pressure gradients compared to the CEP valve was confirmed in this case-matched comparison. However, clinical outcomes in terms of SVD and PVE rates were inferior in this study. Furthermore, the SFS stentless aortic valve showed impaired outcomes regarding mortality in a subgroup of patients who presented with NVE.

A current multicenter study including 565 patients provided with the SFS, reported postoperative transprosthetic pressure gradients of peak/mean 17.7 ± 9.1/ 9.9 ± 5.4 mmHg and a reasonable safety profile with freedom from SVD and re-operation of 90.8% and 87.3% during follow-up [17]. In this analysis the subgroup of patients provided with SAVR for NVE was not further reviewed. While our study confirms the excellent hemodynamic outcomes of the SFS, already known from multiple reports [18; 19], we demonstrated impaired outcomes of patients with NVE treated with the SFS. Reports of surgery for NVE utilizing stentless pericardial valves are scarce and present limited patient numbers with or without group comparison [20; 21]. Explanations for superiority of a stented pericardial valve for AV-NVE are only speculative. Most centers prefer mechanical or stented bioprostheses for AV-NVE surgery [22; 23] and also international guidelines recommend utilization of those prostheses [6] due to best documented results. However, stentless valves may be advantageous in root involvement due to the option of supra-annular implantation or root replacement which is for instance feasible with the Medtronic Freestyle valve. In our institution we followed this approach during the study period of this work. Although we saw more frequent postoperative PVE in the SFS group, the difference between the two groups was not significant and occurrence of more frequent PVE in the SFS group may be due to utilization in severely destroyed aortic annuli. Due to higher re-do rates compared to the utilized stented bioprosthesis and other reports of early SVD [1; 3], this strategy has to be reconsidered.

Patients undergoing re-do surgery of the aortic valve for SVD or PVE present an elevated risk for periprocedural mortality and morbidity [24; 25]. We herein also showed a HR of 7.63 for patients undergoing AV re-do surgery for SVD or PVE. Since the SFS presented a significant higher re-do rate, it should be selected carefully, especially in young patients in which a second operation can be anticipated. On the other hand the effect of re-do surgery in the COX analysis may be due to the small patient number and is worth further investigation. Nevertheless, the SFS should still be considered for small aortic annuli due to the excellent hemodynamics and to avoid patient-prosthesis mismatch. Also, patients with extensive destruction of the aortic annulus in NVE may benefit from the supra-annular implantation technique. Accordingly, SAVR in an infective setting should follow a tailored approach for each individual patient.

## Study limitations

Typical limitations for a retrospective, single-center study with limited patient numbers apply [13]: Patients were not randomized to the respective treatment groups and even though analysis of baseline patient characteristics did not reveal statistically significant inter-group differences results may have been biased by hidden confounders. Furthermore, the choice of the respective bioprosthesis was left to the surgeon’s discretion. Although the SFS was the preferred valve for treatment of NVE during the study period at our institution, this can lead to a certain selection bias.

Moreover, there may be a bias regarding experience with the two different bioprostheses, since the CEP was used more frequent during the study period.

## Conclusions

In this case-matched analysis the SFS stentless pericardial valve presented impaired outcomes in NVE as well as higher rates of re-do surgery, which is connected with a pronounced decrease in freedom from death. Therefore, we are reluctant to recommend utilization of this particular bioprosthesis in patients with infective aortic valve endocarditis or in young patients, due to the anticipated early SVD. The SFS could still be considered for small aortic annuli and severe destruction of the aortic annulus in NVE.

## Abbreviations

ACC: Aortic cross clamp
AV: Aortic valve
CAD: Coronary artery disease
CEP: Carpentier Edwards Perimount
COPD: Chronic obstructive pulmonary disease
CT: Computed tomography
ECC: Extracorporal circulation
EOA: Effective orifice area
IVS: Interventricular septum
LV: Left ventricle
LVEF: Left ventricular ejection fraction
NVE: Native valve endocarditis
NYHA: New York Heart Association
PVE: Prosthetic valve endocarditis
PVL: Paravalvular leakage
SAVR: Surgical aortic valve replacement
SFS: Sorin Freedom Solo
SVD: Structural valve deterioration
TTE: Transthoracic echocardiography
TVL: Transvalvular leakage
